# Supplementary material for: Bacillus-Based Biocontrol Agents Mediate Pathogen Killing by Biodegradable Antimicrobials from Macrolactin Family
Source: Int J Mol Sci. 2025 Nov 19;26(22):11167. doi: 10.3390/ijms262211167 (PMC12653809; doi:10.3390/ijms262211167)
Supplement: Supplementary file 1 [file ijms-26-11167-s001.zip › Supplementary Materials.pdf]

# Supplementary Information

# *Bacillus*-Based Biocontrol Agents Mediate Pathogen Killing by Biodegradable Antimicrobials from Macrolactin Family

**Elena B. Guglya [1], Olga A. Belozerova [1], Anton E. Shikov [2,3], Vera A. Alferova [1], Maria N. Romanenko [2,3], Vladimir K. Chebotar [3], Maria S. Gancheva [2,3], Maria E. Baganova [3], Ekaterina A. Vinogradova [1], Elizaveta A. Marenkova [1], Vladislav A. Lushpa [1,4], Anna A. Baranova [1], Margarita N. Baranova [1], Olga A. Shevtsova [5], Arsen M. Kudzhaev [1], Yuri A. Prokopenko [1], Sergey I. Kovalchuk [1], Dmitrii A. Lukianov [5,6], Kirill S. Antonets [2,3], Anton A. Nizhnikov [2,3] and Stanislav S. Terekhov [1,*]**

[1] Shemyakin-Ovchinnikov Institute of Bioorganic Chemistry, Miklukho-Maklaya 16/10, 117997 Moscow, Russia
[2] Biological Faculty, St. Petersburg State University, 199034 St. Petersburg, Russia
[3] All-Russia Research Institute for Agricultural Microbiology, 196608 St. Petersburg, Russia
[4] Moscow Center for Advanced Studies, Kulakova Str. 20, 123592 Moscow, Russia
[5] Department of Chemistry, Lomonosov Moscow State University, Leninskie Gory, 119992 Moscow, Russia
[6] Center for Molecular and Cellular Biology, 121205 Moscow, Russia
* Correspondence: sterekhoff@gmail.com

# Detailed discussion of the macrolactins' structure elucidation

Analysis of the spectra of compound 1, presumably macrolactin A, revealed the following groups of atoms: one methyl group; six CH2 groups; and fifteen CH groups, three of which are replaced by an oxygen atom. The rest form a double covalent bond with carbon, that is, one quaternary carbon atom, which corresponds to the carboxyl group. Analysis of 1H, HSQC, HMBC, and COSY data allowed for the assembly of a complete molecule corresponding to macrolactin A.

In the case of substance 2, presumably 7-*O*-malonyl macrolactin A, signals from the following groups of atoms are observed: one methyl group; seven $CH_2$ groups; and fifteen CH groups, three of which are replaced by an oxygen atom. The rest form a double covalent bond with carbon, that is, three quaternary carbon atoms, which correspond to carboxyl groups. Analysis of the obtained experimental data allowed us to identify the substance as 7-*O*-malonyl macrolactin A.

In the case of compound 3, presumably 7-*O*-succinyl macrolactin A, signals from the following groups of atoms are observed: one methyl group; eight CH2 groups; and fifteen CH groups, three of which are replaced by an oxygen atom. The rest form a double covalent bond with carbon, that is, three quaternary carbon atoms, which correspond to carboxyl groups. Analysis of the obtained experimental data allowed us to identify the substance as 7-*O*-succinyl macrolactin A.

The structure of the fourth compound was also determined. The NMR spectra showed signals from the following groups: one methyl group; ten CH2 groups; and twelve CH groups, three of which are replaced by an oxygen atom. The rest form a double covalent bond with carbon, that is, four quaternary carbon atoms, three of which correspond to carboxyl groups and one to a carbonyl group. Analysis of all NMR data allowed us to combine the obtained groups into one molecule, which corresponds to 7-*O*-succinyl macrolactin F.

# Supplementary Figures

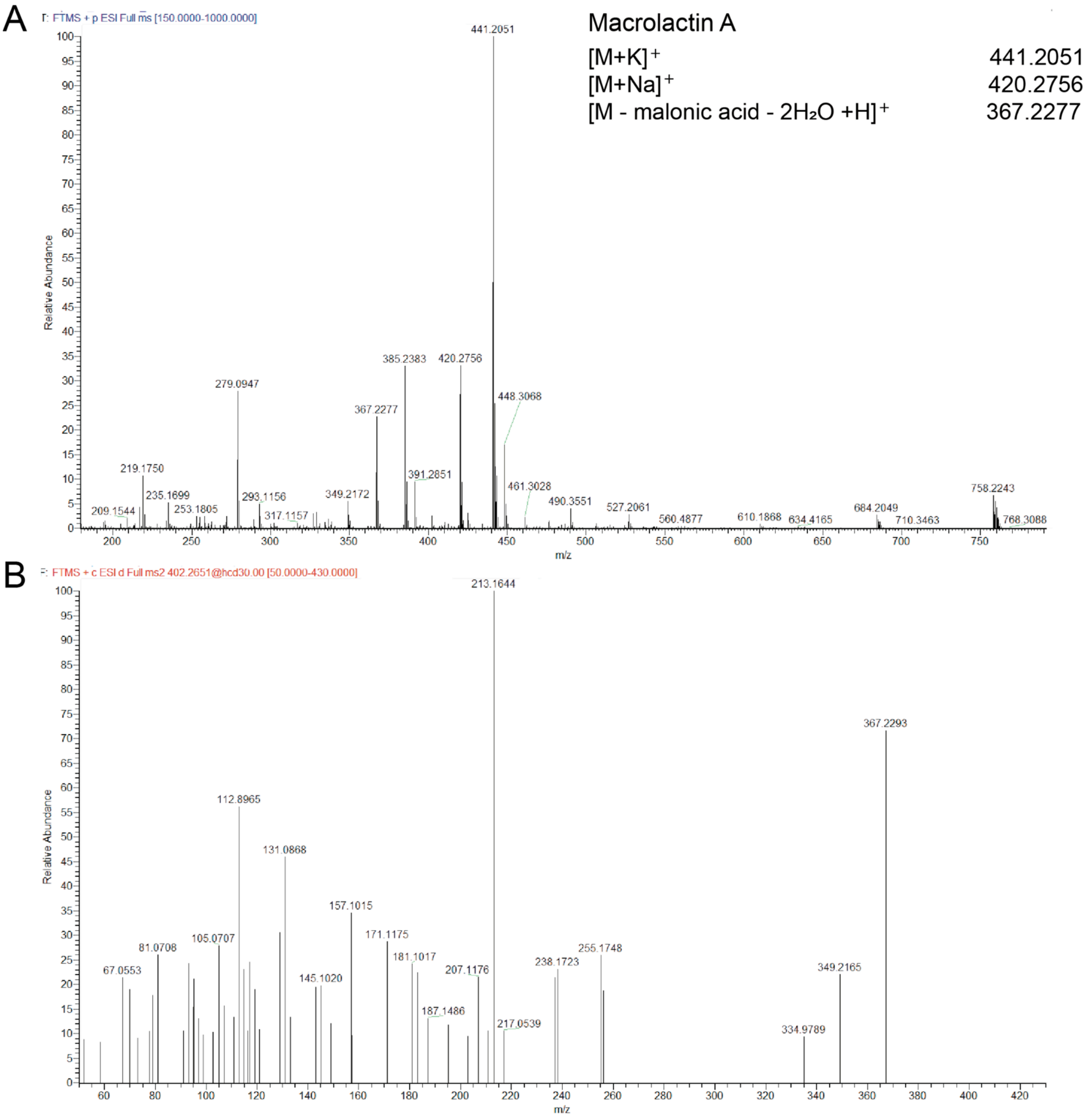


**Figure S1.** (A) Positive ion mode MS1 spectrum of MLN A; (B) HCD mass spectra of parent ion $[M+H]^+$ at *m/z* 402.2651.

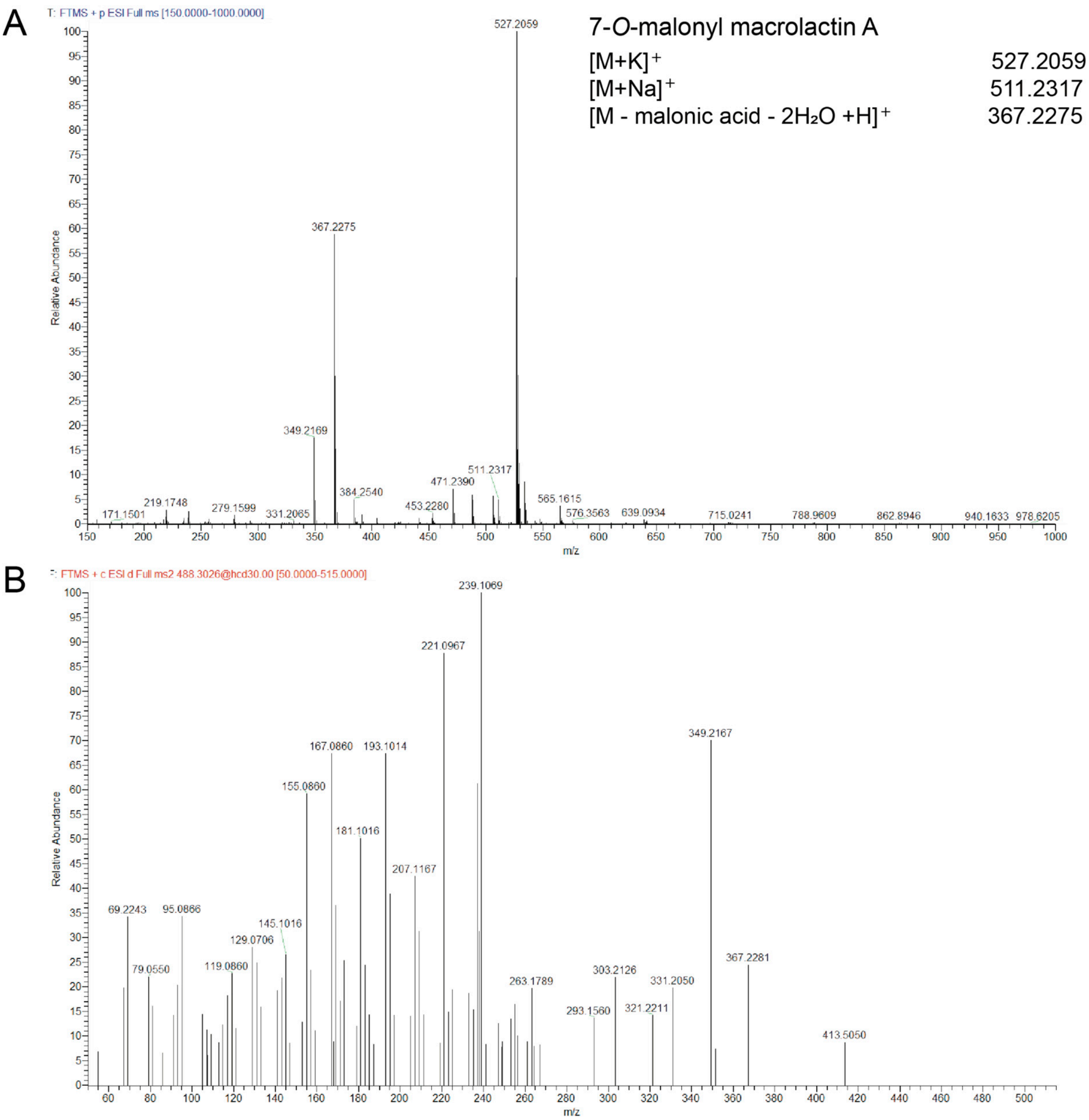


**Figure S2.** (A) Positive ion mode MS1 spectrum of mal-MLN A; (B) HCD mass spectra of parent ion $[M+H]^+$ at *m/z* 488.3026.

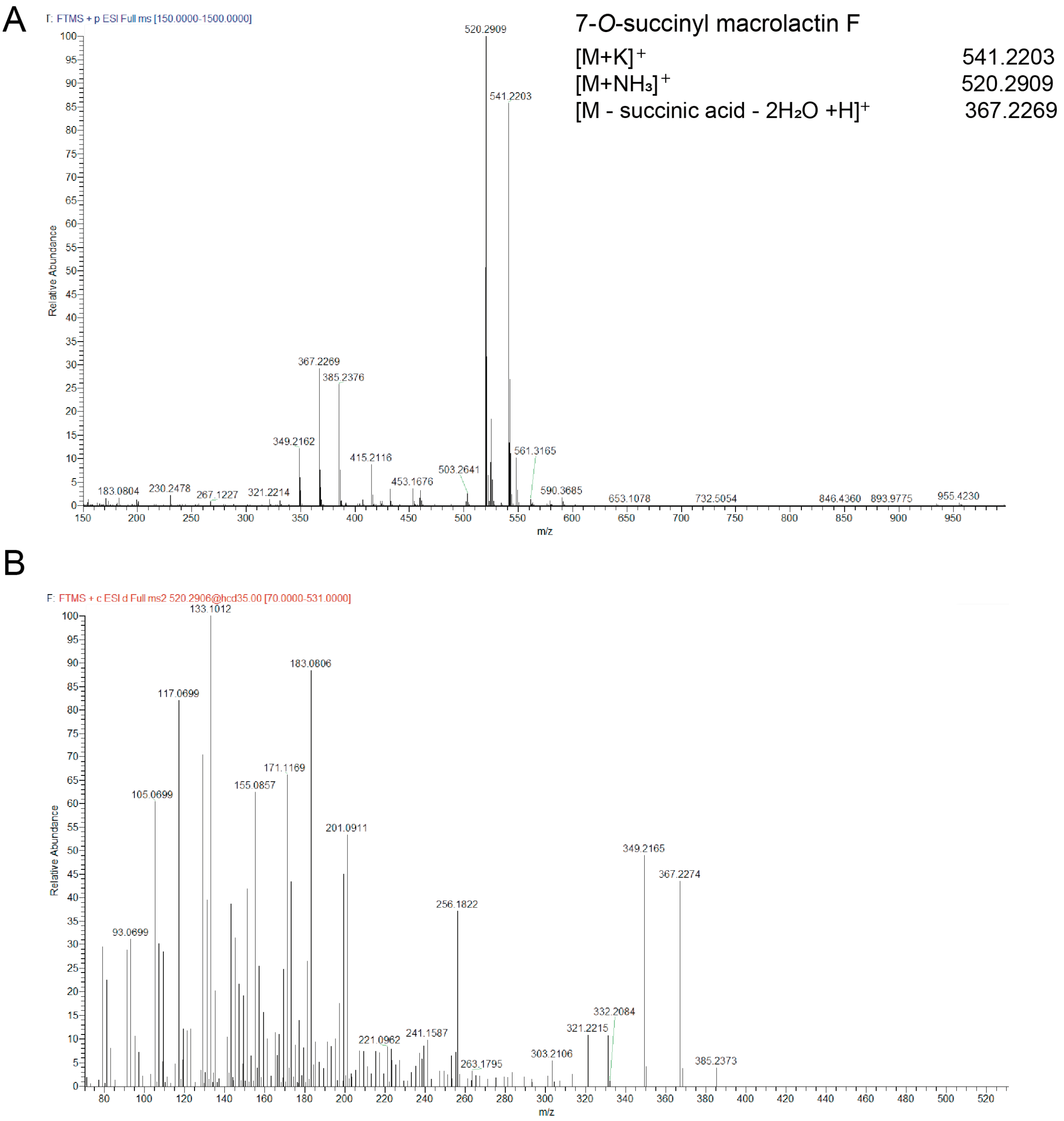


**Figure S3.** (A) Positive ion mode MS1 spectrum of suc-MLN A; (B) HCD mass spectra of parent ion $[M+H]^+$ at *m/z* 502.3196.

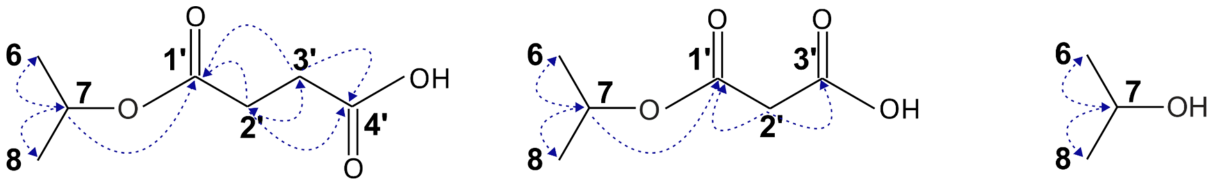


7-*O*-Syccinyl-Macrolactin    7-*O*-Malonyl-Macrolactin    Macrolactin

**Figure S4.** HMBC contacts of the acyl substitutions of the forms of macrolactins observed in the NMR spectra. The blue arrows show the observed HMBC contacts in the NMR spectra.

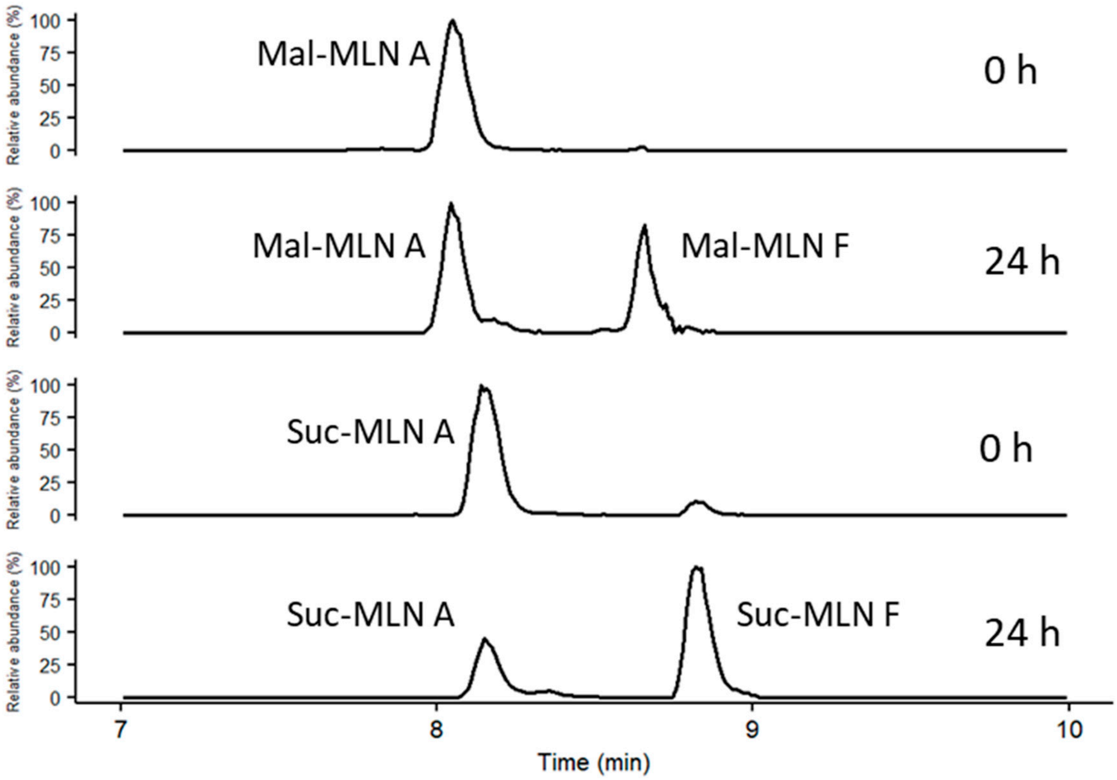


**Figure S5.** LC–MS extracted ion chromatograms (EICs) of broth samples after 0 and 24 h of incubation with macrolactins, monitored for $[M+K]^+$ adducts of Mal-MLN A (m/z 527.2047), Suc-MLN A (m/z 541.2204), and MLN A (m/z 441.2043). Intensities are shown relative to the most intense signal.

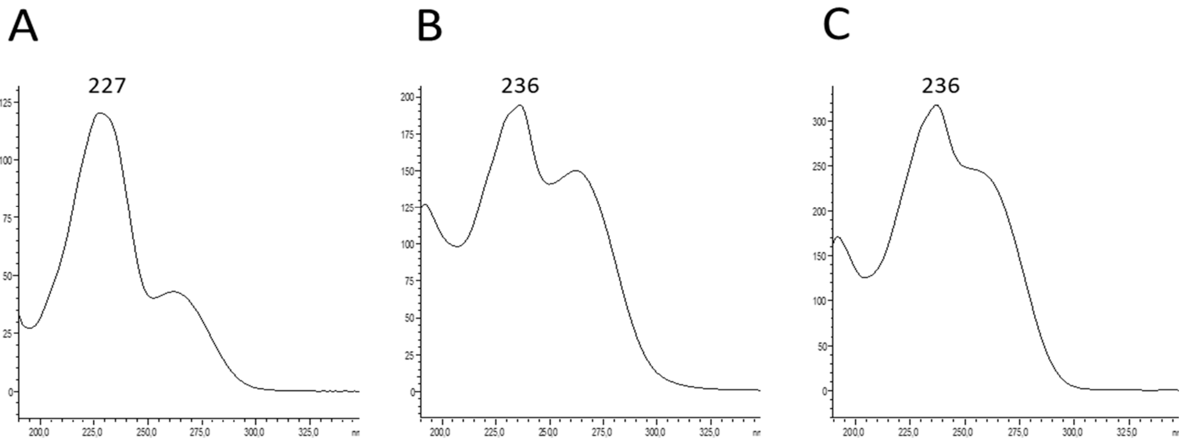


**Figure S6.** UV spectra of the macrolactins in Figure 3. (A) Spectra of MLN A, mal-MLN A, and suc-MLN A. (B) Spectrum of MLN F. (C) Spectrum of suc-MLN F and mal-MLN F.

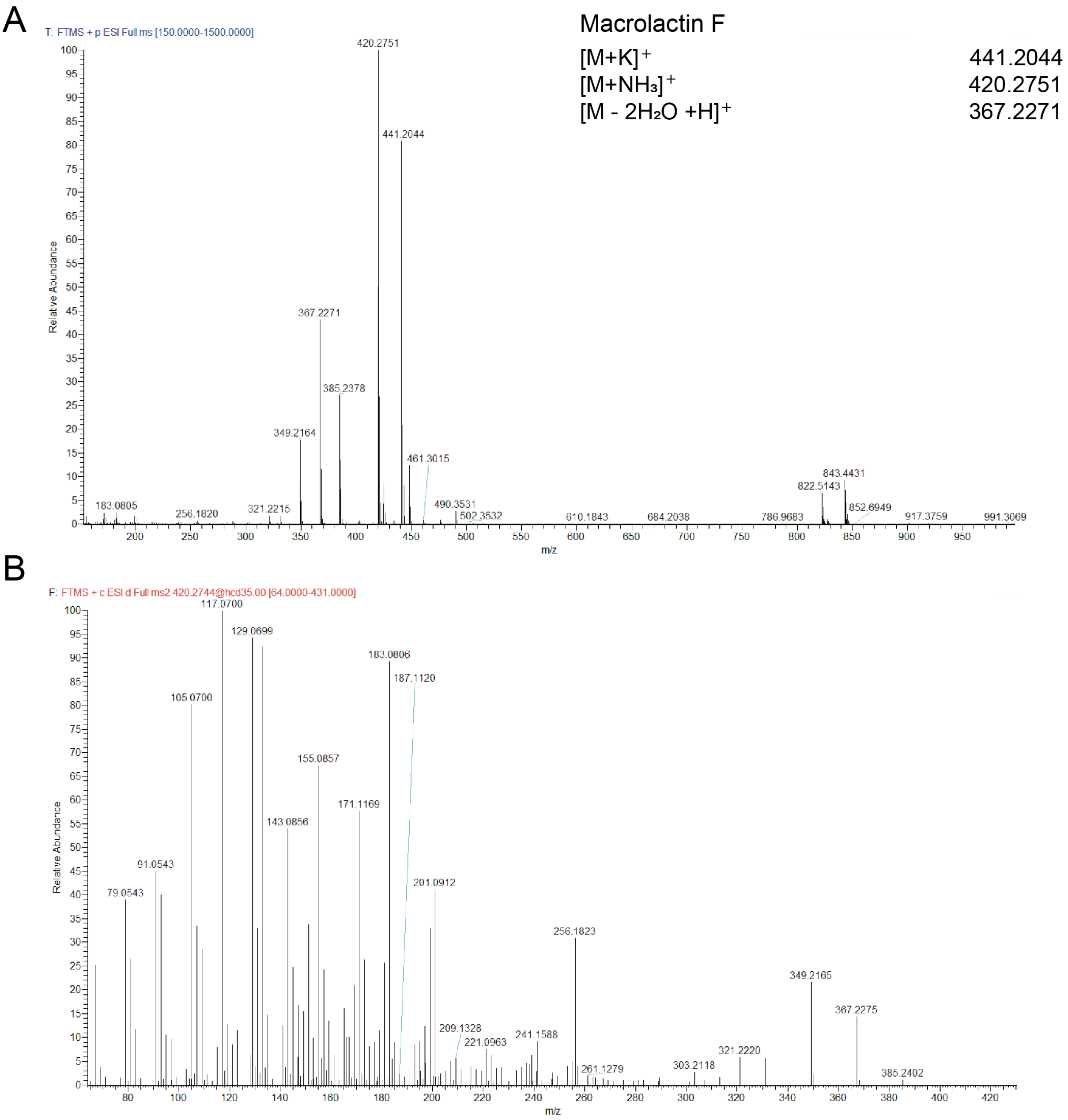


**Figure S7.** (A) Positive ion mode MS1 spectrum of MLN F; (B) HCD mass spectra of parent ion $[M+H]^+$ at *m/z* 402.2651.

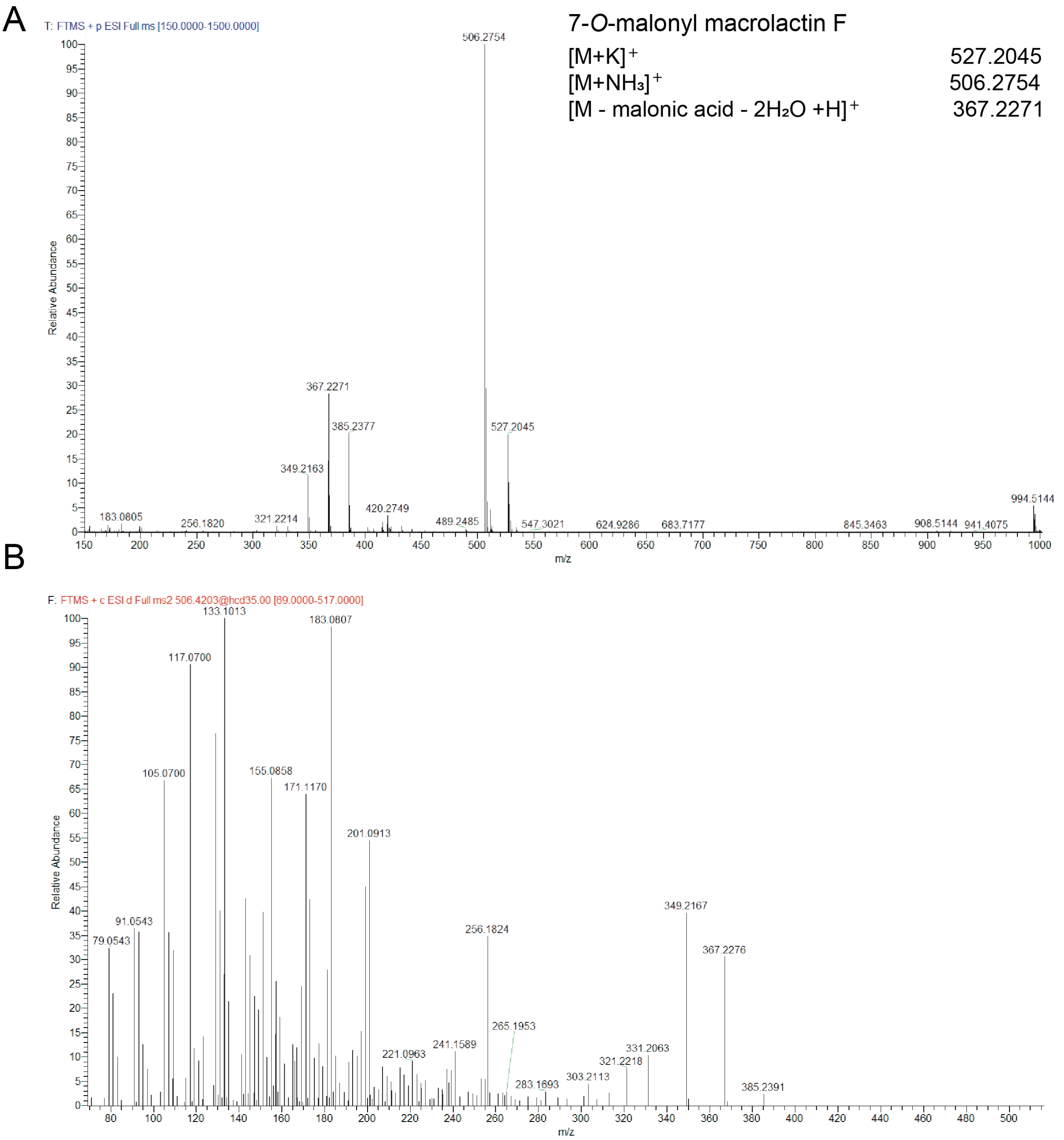


**Figure S8.** (A) Positive ion mode MS1 spectrum of mal-MLN F; (B) HCD mass spectra of parent ion $[M+H]^+$ at *m/z* 488.3026.

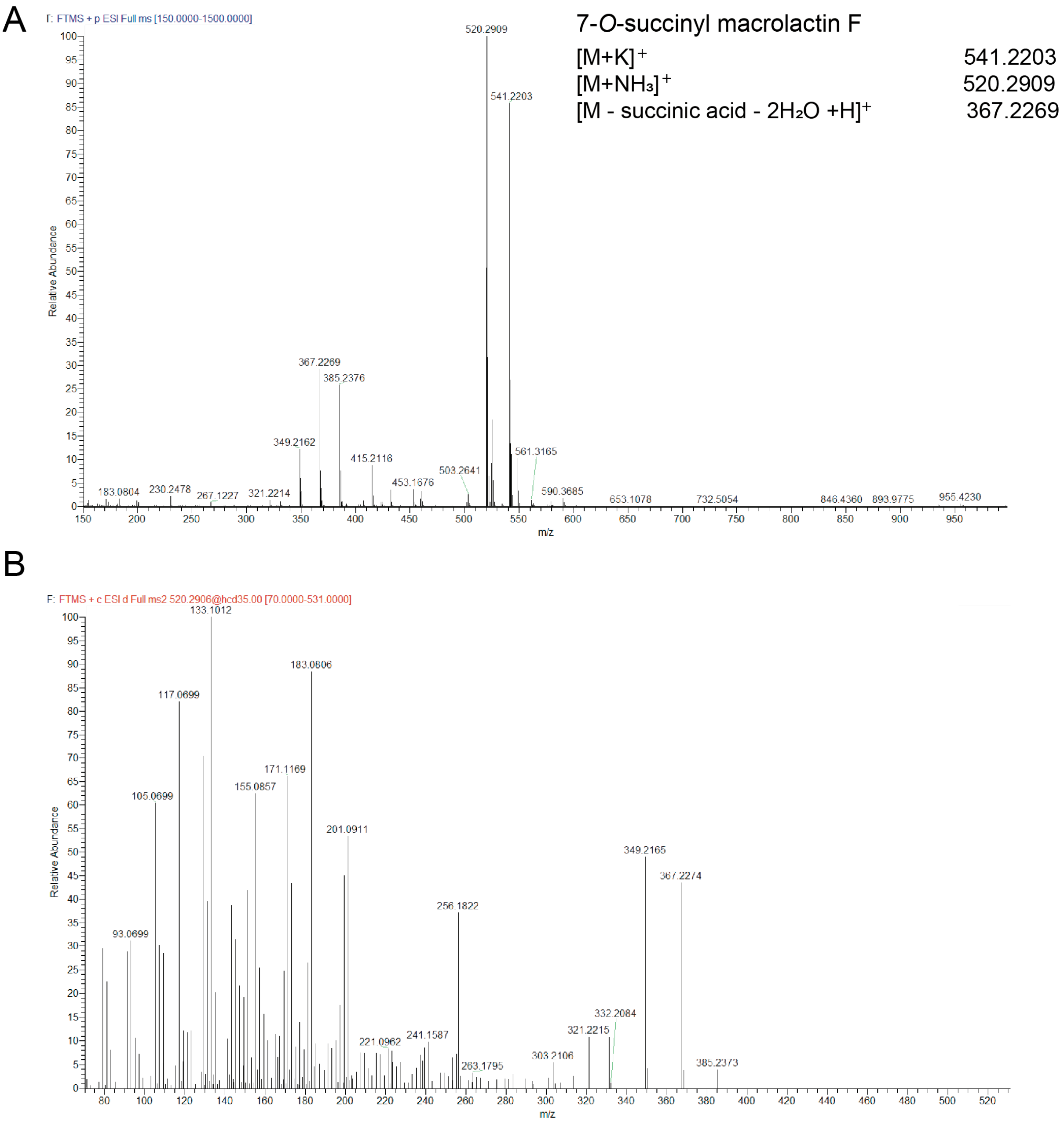


**Figure S9.** (A) Positive ion mode MS1 spectrum of suc-MLN F; (B) HCD mass spectra of parent ion $[M+H]^+$ at *m/z* 502.3196.

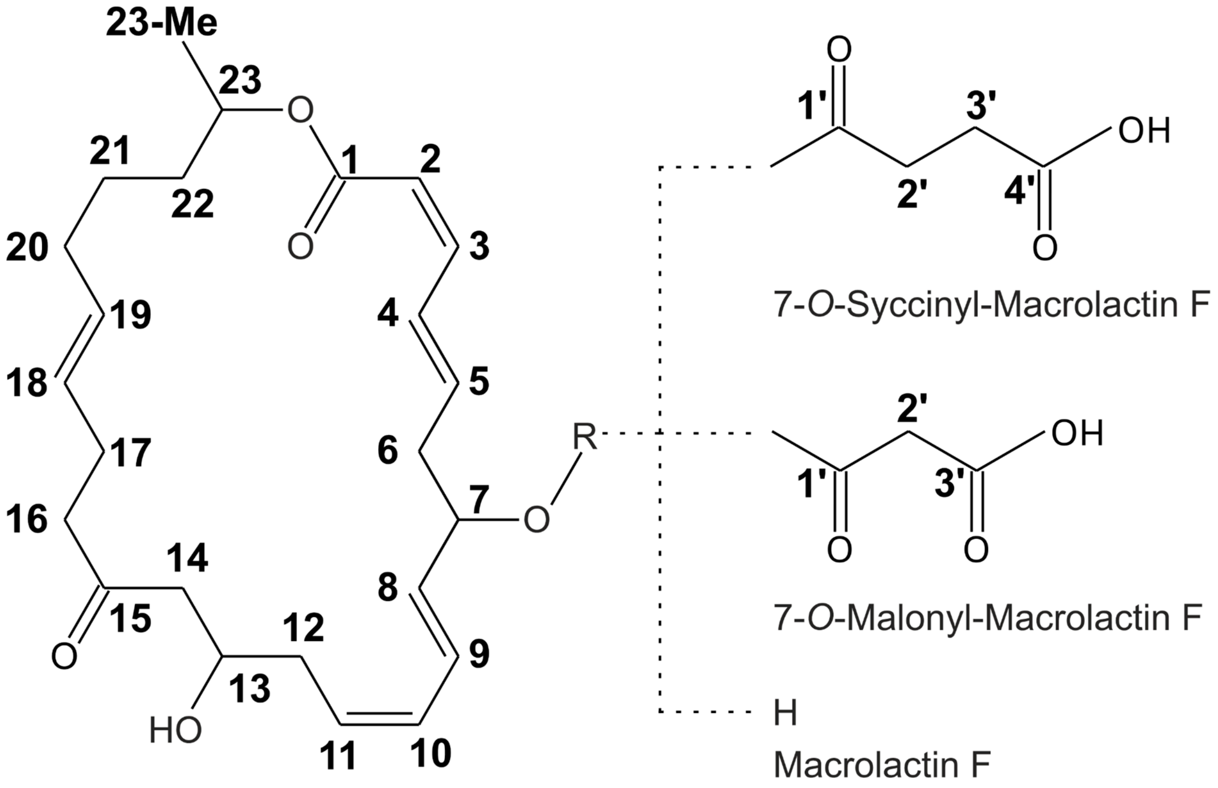


**Figure S10.** Structures of macrolactins according to NMR data. The designation R is introduced for the variable part of the molecules (see also Table S2).

# Supplementary Tables

**Table S1.** Maximal inhibiting dilution of *B. velezensis* K-3618 culture media obtained for BHI, 2YT, and SYC growth media against *S. aureus* and *E. coli* Δ*lptD* bacterial cells.

| Target bacterium | Cultivation medium | Cultivation time, days | | |
|---|---|---|---|---|
| | | 1 | 2 | 4 |
| | | Maximum inhibitory dilution | | |
| *S. aureus* | BHI | 128 | 128 | 128 |
| | 2YT | 128 | 128 | 128 |
| | SYC | 64 | 16 | 8 |
| *E. coli* Δ *lptd* | BHI | 2 | 2 | 2 |
| | 2YT | 4 | 2 | - |
| | SYC | 32 | 8 | - |

**Table S2.** Assignment of NMR signals of macrolactin A and its derivatives. The following abbreviations have been introduced: MLN A—macrolactin A, mal-MLN A—7-*O*-malonyl macrolactin A, suc-MLN—7-*O*-succinyl macrolactin A.

| | | MLN A | | Mal-MLN A | | Suc-MLN A | |
|---|---|---|---|---|---|---|---|
| | | δ, ppm | | δ, ppm | | δ, ppm | |
| № $^{13}$C | group | 13C | 1H | 13C | 1H | 13C | 1H |
| 1 | -C=O | 166.25 | | 166.45 | | 166.75 | |
| 2 | -CH= | 118.09 | 5.618 | 118.20 | 5.630 | 118.2 | 5.67 |
| 3 | -CH= | 142.76 | 6.561 | 141.70 | 6.561 | 141.9 | 6.561 |
| 4 | -CH= | 129.86 | 7.249 | 130.34 | 7.131 | 130.2 | 7.177 |
| 5 | -CH= | 139.24 | 5.921 | 137.24 | 5.987 | 137.7 | 6.022 |
| 6 | -CH2- | 41.48 | 2.482 | 39.19 | 2.633 | 39.31 | 2.646 |
| 7 | -CH-O | 71.29 | 4.361 | 72.70 | 5.693 | 72.22 | 5.15 |
| 8 | -CH= | 132.80 | 5.631 | 129.86 | 5.681 | 131.9 | 5.773 |
| 9 | -CH= | 125.40 | 6.576 | 125.61 | 6.788 | 124.41 | 6.721 |
| 10 | -CH= | 130.50 | 6.142 | 129.68 | 6.098 | 130.33 | 6.081 |
| 11 | -CH= | 127.80 | 5.551 | 127.82 | 5.411 | 127.7 | 5.43 |
| 12 | -CH2- | 35.39 | 2.429, 2.499 | 34.26 | 2.996, 2.406 | 34.34 | 3.1 / 2.48 |
| 13 | -CH-OH | 69.63 | 4.001 | 70.53 | 4.130 | 70.79 | 4.21 |
| 14 | -CH2- | 40.92 | 1.777 | 38.59 | 1.626, 1888 | 38.71 | 1.935 / 1.71 |
| 15 | -CH-OH | 70.41 | 4.541 | 72.30 | 5.721 | 70.27 | 4.655 |
| 16 | -CH= | 132.71 | 5.627 | 130.92 | 5.711 | 131.1 | 5.771 |
| 17 | -CH= | 130.69 | 6.207 | 131.81 | 6.241 | 131.72 | 6.17 |
| 18 | -CH= | 129.87 | 6.040 | 130.34 | 6.028 | 130.4 | 6.136 |
| 19 | -CH= | 135.03 | 5.689 | 135.71 | 5.629 | 135.61 | 5.681 |
| 20 | -CH2- | 32.04 | 2.117, 2.193 | 31.90 | 2.234, 2.085 | 31.81 | 2.285 / 2.163 |
| 21 | -CH2- | 24.47 | 1.521 | 24.30 | 1.559 | 24.25 | 1.577 |

| | | | | | | | |
|---|---|---|---|---|---|---|---|
| 22 | -CH2- | 35.11 | 1.577, 1.681 | 34.72 | 1.678, 1.581 | 34.73 | 1.734 / 1.638 |
| 23 | -CHO- | 70.84 | 5.157 | 71.75 | 5.006 | 72.57 | 5.077 |
| 23-Me | -CH3 | 19.94 | 1.291 | 20.01 | 1.311 | 20.04 | 1.356 |
| 1’ | -COO | | | 166.2 | | 170.96 | |
| 2’ | -CH2- | | | 42.17 | 3.384, 3.492 | 28.665 | 2.795 / 2.672 |
| 3’ | -COOH/CH2- | | | 169.2 | | 29.66 | 2.69 / 2.702 |
| 4’ | -COOH | | | | | 174.3 | |

**Table S3.** Assignment of NMR signals of macrolactin F and its derivatives. The following abbreviations have been introduced: MLN F—macrolactin F; suc-MLN F—7-*O*-succinyl macrolactin F.

| | | MLN F | | Suc-MLN F | |
|---|---|---|---|---|---|
| | | δ, ppm | | δ, ppm | |
| № $^{13}C$ | group | 13C | 1H | 13C | 1H |
| 1 | -C=O | 166.60 | | 165.9 | |
| 2 | -CH= | 118.00 | 5.612 | 117.08 | 5.436 |
| 3 | -CH= | 142.81 | 6.532 | 143.24 | 6.635 |
| 4 | -CH= | 128.37 | 7.280 | 129.71 | 7.302 |
| 5 | -CH= | 137.71 | 5.952 | 138.44 | 6.094 |
| 6 | -CH2- | 41.16 | 2.508 | 37.91 | 2.592 |
| 7 | -CH-OH | 71.38 | 4.365 | 73.14 | 5.436 |
| 8 | -CH= | 130.34 | 5.644 | 130.57 | 5.452 |
| 9 | -CH= | 125.53 | 6.507 | 127.25 | 6.552 |
| 10 | -CH= | 130.39 | 6.085 | 129.90 | 6.121 |
| 11 | -CH= | 131.80 | 5.431 | 128.08 | 5.576 |
| 12 | -CH2- | 47.61 | 2.551, 2.630 | 34.63 | 2.420 |
| 13 | -CH-OH | 67.61 | 4.191 | 67.37 | 4.110 |
| 14 | -CH2- | 38.36 | 2.565 | 48.32 | 2.595 |
| 15 | -C=O | 212.10 | | 210.6 | |
| 16 | -CH2- | 43.72 | 2.495 | 42.93 | 2.499 |
| 17 | -CH2- | 26.71 | 2.290 | 26.52 | 2.242 |
| 18 | -CH= | 130.98 | 5.440 | 128.91 | 5.448 |
| 19 | -CH= | 128.81 | 5.399 | 130.57 | 5.442 |
| 20 | -CH2- | 31.97 | 1.981, 2.064 | 31.68 | 2.005 2.079 |
| 21 | -CH2- | 24.91 | 1.449 | 27.71 | 1.443 |
| 22 | -CH2- | 35.08 | 1.529, | 34.80 | 1.562 |

| | | | | | |
|---|---|---|---|---|---|
| | | | 1.661 | | 1.667 |
| 23 | -CHO- | 70.87 | 5.012 | 70.62 | 5.016 |
| 23-Me | -CH3 | 19.92 | 1.271 | 18.87 | 1.258 |
| 1’ | -COO | | | 172.15 | |
| 2’ | -CH2- | | | 29.36 | 2.617<br>2.648 |
| 3’ | -<br>COOH/CH2<br>- | | | 29.02 | 2.599<br>2.630 |
| 4’ | -COOH | | | 175.25 | |
